# Phytolith analysis for differentiating between broomcorn millet (*Panicum miliaceum*) and its weed/feral type (*Panicum ruderale*)

**DOI:** 10.1038/s41598-018-31467-6

**Published:** 2018-08-29

**Authors:** Jianping Zhang, Houyuan Lu, Minxuan Liu, Xianmin Diao, Konglan Shao, Naiqin Wu

**Affiliations:** 1 Key Laboratory of Cenozoic Geology and Environment, Institute of Geology and Geophysics, Chinese Academy of Sciences, Beijing, 100029 China; 2 Center for Excellence in Tibetan Plateau Earth Science, Chinese Academy of Sciences, Beijing, 100101 China; 3 University of Chinese Academy of Sciences, Beijing, China; 4 Institute of Crop Sciences, Chinese Academy of Agricultural Sciences, Beijing, 100081 China

## Abstract

Domestication of broomcorn millet (*Panicum miliaceum*) is one of the most significant events in prehistoric East Asia, providing sufficient food supply for the explosive growth of Neolithic populations and the transition into complex societies. However, to date, the process of broomcorn millet domestication is still largely unknown, partly due to the lack of clear diagnostic tools for distinguishing between millet and its related wild grasses in archaeological samples. Here, we examined the percentage of silicified epidermal long-cell undulated patterns in the glume and palea from inflorescence bracts in 21 modern varieties of broomcorn millet and 12 weed/feral-type *Panicum ruderale* collected across northern China. Our results show that the percentage of ηIII patterns in domesticated broomcorn millet (23.0% ± 5.9%; *n* = 63) is about 10% higher than in *P*. *ruderale* (10.8% ± 5.8%; *n* = 36), with quartiles of 17.2–28.3% and 5.1–15.5%, respectively. Owing to the increase in ηIII pattern percentage correlates significantly with a decrease in the grain length/width ratio, in the absence of exact wild ancestors of broomcorn millet, the characterization of phytolith differences between *P*. *ruderale* and *P*. *miliaceum* thus becomes an alternative approach to provide insight into origin of broomcorn millet.

## Introduction

Broomcorn millet (*Panicum miliaceum*; a tetraploid cereal, 2*n* = 4*x* = 36) is one of the oldest staple cereals in East Asia, dating back to the beginning of the Holocene and used across the entire Eurasian continent prior to the popularity of rice and wheat; it has also emerged as one of the most aggressive grass weeds in North America and Canada^1–3^. Understanding the process of broomcorn millet domestication is key to our comprehension of the rise of agriculture in this vast region, especially in East Asia. However, to date, the questions of where, when and how the cereal transitioned from simple gathering to domestication remain largely unanswered due to the lack of definite identifiable features distinguishing domesticated broomcorn millet and its wild ancestor.

*Panicum miliaceum* subsp. *ruderale* (Kitag) Tzvel, or *Panicum ruderale* (Kitag.) Chang comb. Nov. (2*n* = 36), exhibits a widespread distribution across a region spanning from West Asia to China^4^. Previous research indicates that it could possibly represent either a wild ancestor or a weed/feral form of *P*. *miliaceum*^5,6^. The morphological characteristics of this form are largely similar to domesticated broomcorn millet, except for the dark pericarp color, shorter stature, more sparsely opened and shattered panicles, fewer spikelets per panicle, more branches, and smaller seeds^5,6^.

Distinguishing between wild ancestors and their respective domesticated varieties is essential for understanding crop domestication history^7^. To date, little attention has been paid to uncovering the domestication history of broomcorn millet and its related wild grasses. The only obvious difference between broomcorn millet and *P*. *ruderale* that can be readily detected is the seed size. However, the carbonized seeds from archaeobotanical remains are difficult to distinguish due to their tiny grain size, delicate chaff, and similar shape. In addition, diagnostic features are often lost when seeds are oxidized into granules or ash, or broken into pieces in early Holocene archaeobotanical assemblages^8,9^.

Since phytoliths are silica casts of plant cells created within and between cells of living plants tissues that can remain in sediments long after the living tissue has decayed, phytolith analysis makes the identification of decayed plant remains from archaeological samples possible and exact, i.e., rice, maize, foxtail millet and broomcorn millet can be identified according to shape, size, and other anatomical features^10–13^. In recent years, distinguishing wild and domesticated species in phytoliths occurring within archaeological residues has become a topic of great scientific interest^7^. However, owing to the highly similar phytolith shape within a given genus, it is not adequate to use morphological features alone to distinguish crop plants from their wild ancestors. The development of phytolith morphometry has enabled such distinctions to be made, and morphometry-based approaches are increasingly used to reveal crop domestication processes. These approaches include characterizing the fish-like scales of bulliform phytoliths of rice and the inflorescence phytoliths of millet and wheat^14–20^.

In the present study, we investigated 21 landraces of modern broomcorn millet and 12 specimens of *P*. *ruderale* from eight provinces cross north China in order to determine whether analyzing the phytoliths of inflorescence bracts can be used as an effective tool for discriminating broomcorn millet from its possible wild ancestor or weed/feral type, *P*. *ruderale*. Our findings provide a potential method to investigate the process of broomcorn millet domestication.

## Materials and Methods

**Figure 1:**
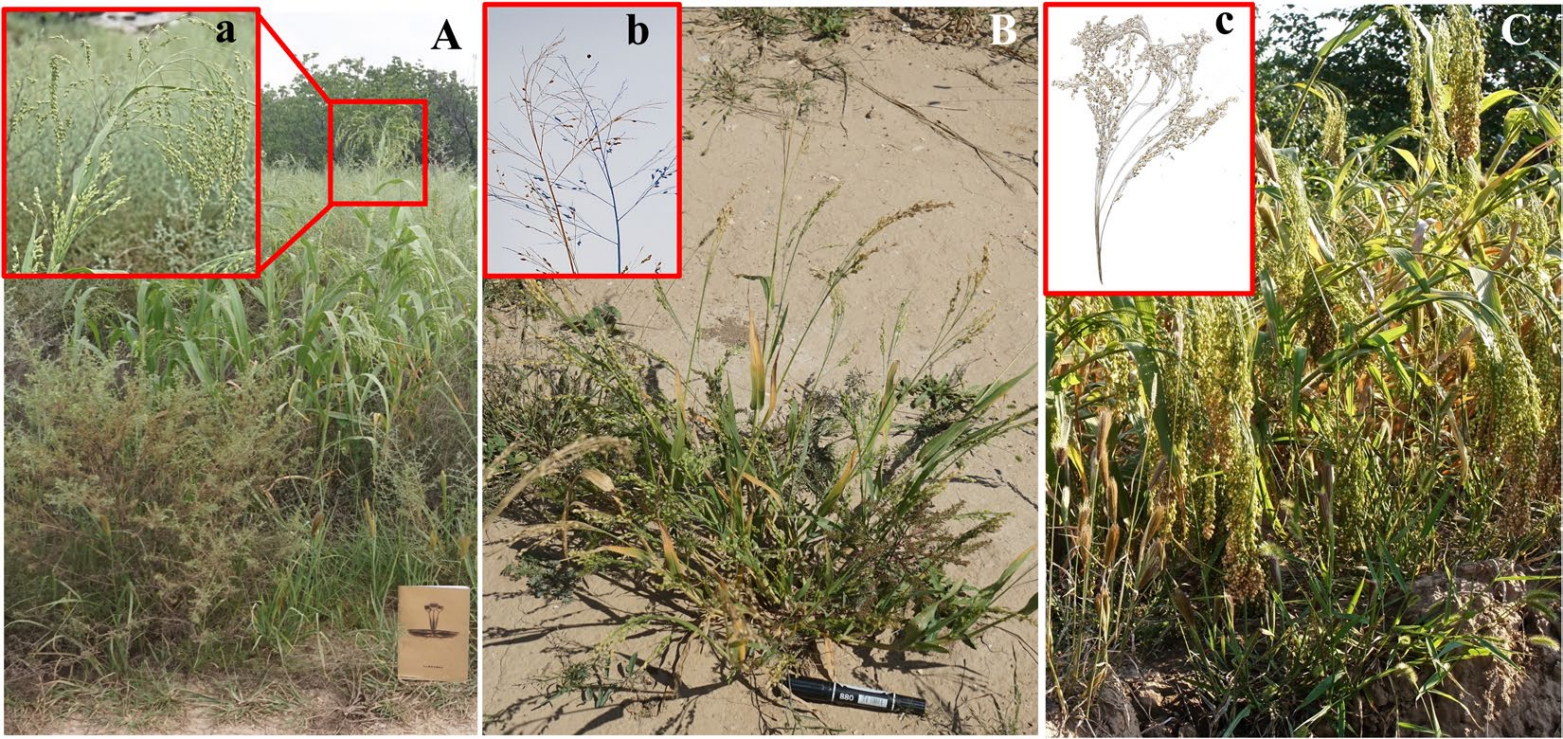
Vegetative traits. (**A**) *Panicum ruderale* with longer stature, loose panicle (a), (**B**) *Panicum ruderale* with shorter stature, more basal branches, loose panicle (b), (**C**) *Panicum miliaceum* with close panicle (c).

**Figure 2:**
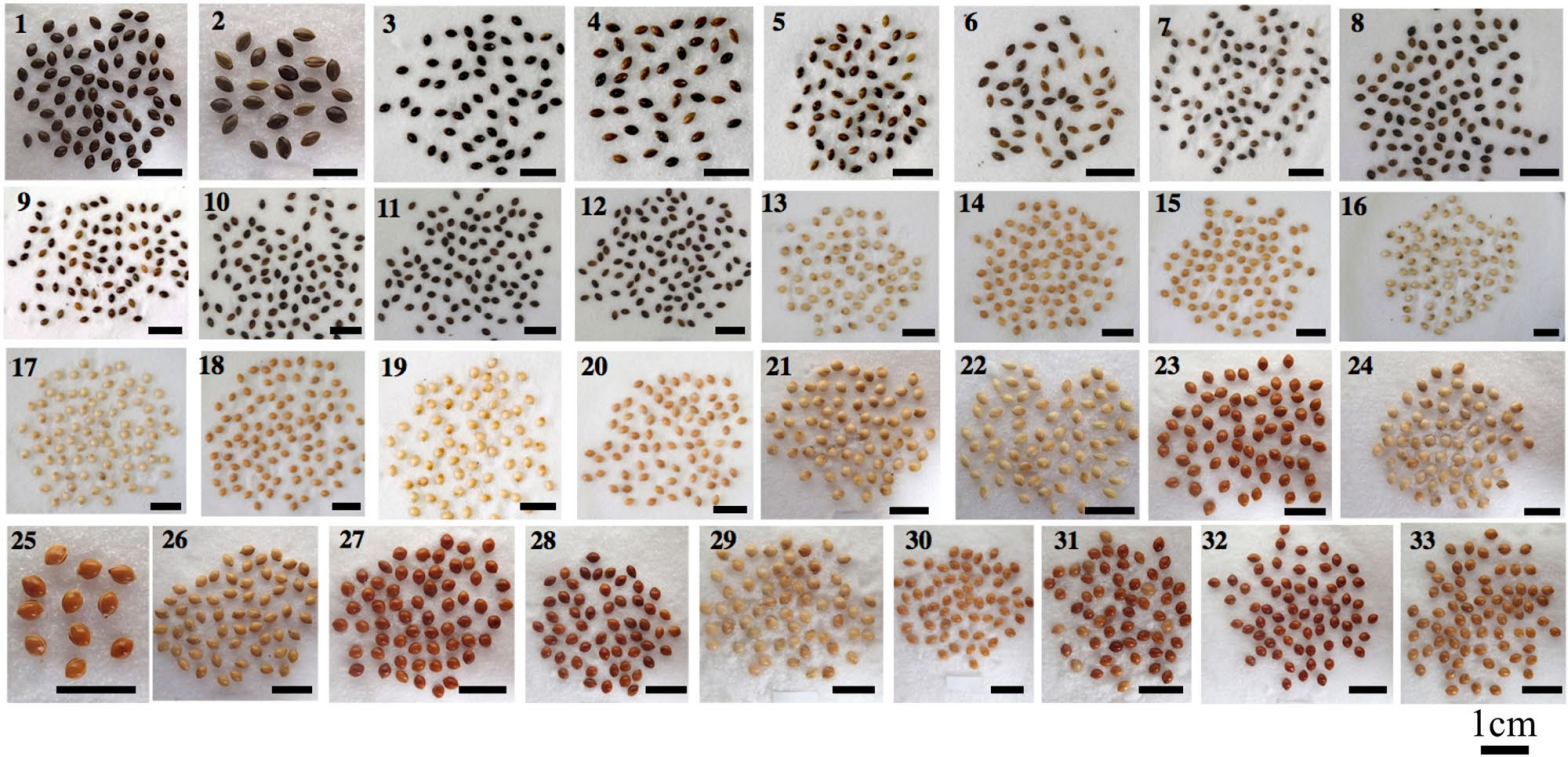
Photographs of tested cereal grains.(1–12) *Panicum ruderale*, (13–33) *Panicum miliaceum*.

*P*. *ruderale* features dark grey or dark brown pericarps, stands 40 to 100 cm tall, has a large number of branches, and forms a loose panicle, whereas *P*. *miliaceum* (broomcorn millet) has white, yellow, brown or red pericarps (accounting for over 90% of the total), stands 100 to 150 cm tall, with close, loose, or lateral panicles and less branches^5,21^ (Fig. 1). In this study, we selected 12 modern *P*. *ruderale*, all of which have dark pericarp coloring, and 21 landraces of modern *P*. *miliaceum* with light pericarps. For *P*. *ruderale*, nos. 1 to 6 were collected in 2012 and 2017 from field investigations conducted near roadsides and abandoned fields in Yangyuan County, Hebei Province, northern China, and nos. 7 to 12 were collected from Institute of Crop Sciences, Chinese Academy of Agricultural Sciences (ICSCAAS). Landraces of modern *P*. *miliaceum* were collected from ICSCAAS (nos. 13 to 20) and the Institute of Geology and Geophysics, Chinese Academy of Sciences (IGGCAS; nos. 21 to 33). Notably, nos. 21 and 28 were collected in the same location in Yangyuan County where *P*. *ruderale* specimens were collected. For detailed descriptions of the plants, see Table 1 and Fig. 2. For comparison of wild relatives of *P*. *ruderale*, we collected one sample of *Panicum repens* from IGGCAS.

**Table 1:** Details of the tested plants.

No.	Source	Species	ICSCAAS no.	Breed name	Province
1	IGGCAS	*P*. *ruderale*	/	/	Hebei
2	IGGCAS	*P*. *ruderale*	/	/	Hebei
3	IGGCAS	*P*. *ruderale*	/	/	Hebei
4	IGGCAS	*P*. *ruderale*	/	/	Hebei
5	IGGCAS	*P*. *ruderale*	/	/	Hebei
6	IGGCAS	*P*. *ruderale*	/	/	Hebei
7	ICSCAAS	*P*. *ruderale*	6761	/	Inner Mongolia
8	ICSCAAS	*P*. *ruderale*	6762	/	Shanxi
9	ICSCAAS	*P*. *ruderale*	6763	/	Inner Mongolia
10	ICSCAAS	*P*. *ruderale*	6764	/	Inner Mongolia
11	ICSCAAS	*P*. *ruderale*	6766	/	Xinjiang
12	ICSCAAS	*P*. *ruderale*	6767	/	Gansu
13	ICSCAAS	*P*. *miliaceum*	481	Zhalantunbaishuzi	Inner Mongolia
14	ICSCAAS	*P*. *miliaceum*	529	Linxihuangshuzi	Inner Mongolia
15	ICSCAAS	*P*. *miliaceum*	702	Huinonghuangnianshu	Ningxia
16	ICSCAAS	*P*. *miliaceum*	767	Erqingshu	Hebei
17	ICSCAAS	*P*. *miliaceum*	1055	Jiguanshu	Shanxi
18	ICSCAAS	*P*. *miliaceum*	1648	Jitouruanmi	Shaanxi
19	ICSCAAS	*P*. *miliaceum*	1655	Bairuanzhoumi	Shaanxi
20	ICSCAAS	*P*. *miliaceum*	3065	Huangmi	Xinjiang
21	IGGCAS	*P*. *miliaceum*	/	/	Hebei
22	IGGCAS	*P*. *miliaceum*	/	/	Hebei
23	IGGCAS	*P*. *miliaceum*	/	/	Shanxi
24	IGGCAS	*P*. *miliaceum*	/	/	Gansu
25	IGGCAS	*P*. *miliaceum*	/	/	/
26	IGGCAS	*P*. *miliaceum*	/	/	Gansu
27	IGGCAS	*P*. *miliaceum*	/	/	Heilongjiang
28	IGGCAS	*P*. *miliaceum*	/	/	Hebei
29	IGGCAS	*P*. *miliaceum*	/	/	Gansu
30	IGGCAS	*P*. *miliaceum*	/	/	Gansu
31	IGGCAS	*P*. *miliaceum*	/	/	Gansu
32	IGGCAS	*P*. *miliaceum*	/	/	Gansu
33	IGGCAS	*P*. *miliaceum*	/	/	Gansu

For phytolith statistics, it is essential that all samples are subjected to identical physical treatment; thus, we applied the same method to all samples. We took 10 seeds from each plant specimen, and (i) cleaned the whole seeds with distilled water to remove adhering particles, (ii) placed all samples in 20 ml of saturated nitric acid and placed in a boiling water bath for 50 min to oxidize organic materials completely, (iii) added distilled water and centrifuged at 2500 rpm. for 5 min, (iv) decanted and rinsed with absolute ethanol and centrifuged at 2500 rpm. for 5 min (twice), and (v) transferred the resulting phytoliths to storage vials. The residual subsamples were mounted onto microscopic slides in Canada Balsam medium for observation.

The number of ηI, II, and III patterns were counted three times for each plant specimen. For the first count, we randomly selected 20 pieces of phytolith regardless of the size of the piece; for the second count, we selected 20 pieces of phytolith between 125 μm and 375 μm regardless of how many single undulated patterns occurred in one piece; for the final count, we selected 20 phytolith pieces sized not only between 125 μm and 375 μm, but also containing more than 30 single undulated patterns in each piece. For each count in each specimen, we calculated the percentage of ηIII from more than 1,000 single undulated patterns. Finally, we calculated 36 total values (12 specimens ×3 counts) representing the percentage of ηIII in *P*. *ruderale*, and 63 (21 specimens ×3 counts) values in domesticated millet, by counting over 160,000 single undulating patterns.

**Figure 3:**
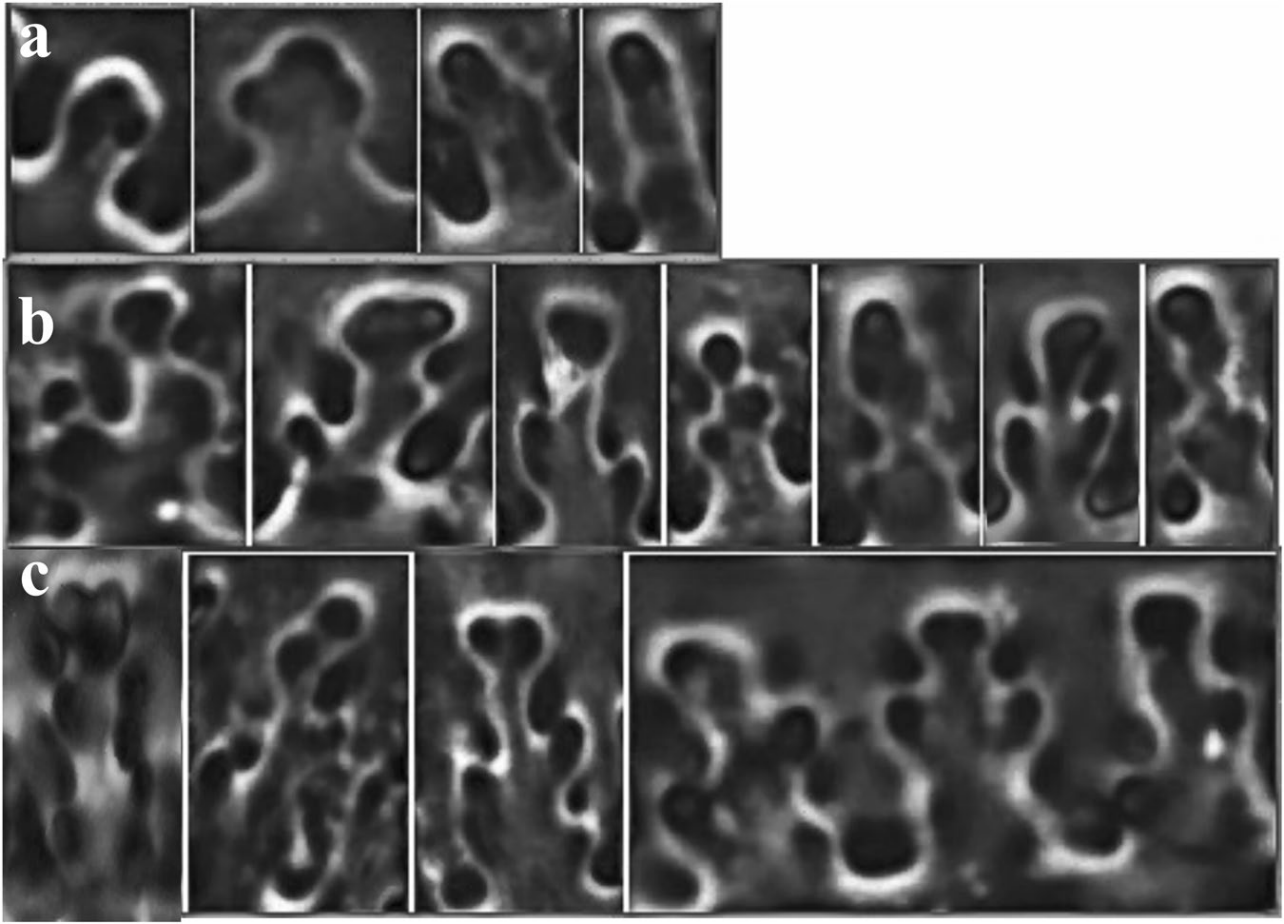
Morphology of ηI (**a**), ηII (**b**) and ηIII (**c**) pattern of epidermal long cells.

Phytoliths were observed by phase-contrast light microscopy at 400× magnification. The identification of ηI, ηII, and ηIII was aided by reference materials described in Lu *et al*.^13^. To unify the identification criteria, we further specified the undulating patterns of ηI, ηII, and ηIII based on reference materials in Lu *et al*.^13^ (Fig. 3). As Fig. 3 shows, according to the numbers of sub-branches along the two sides of main branches, we classify three sub-types as follows: ηI exhibits only one main branch or the top part of the main branch is symmetrically expanded; the ηII subtype excludes the top-expanded branches, and if present, only one sub-branch occurs in one side of the main branch; ηIII have more than one sub-branch in one side of the main branch – usually two or three. Notably, the epidermal long cells within the joint part were avoided when counting the undulating patterns, because the undulating patterns around the joint part were too sinuous to allow correct identification.

Morphometric measurements were performed on an average of 60 grains for each sample. To minimize measurement error, we placed the grains on a soft salt layer in the culture dish, ensuring that the widest profile of the grains faced upward. Specifically, grain width (W) and length (L) were measured. The ratio of L/W was calculated. Descriptive statistics and Pearson tests on the quantitative data were performed.

## Results

**Figure 4:**
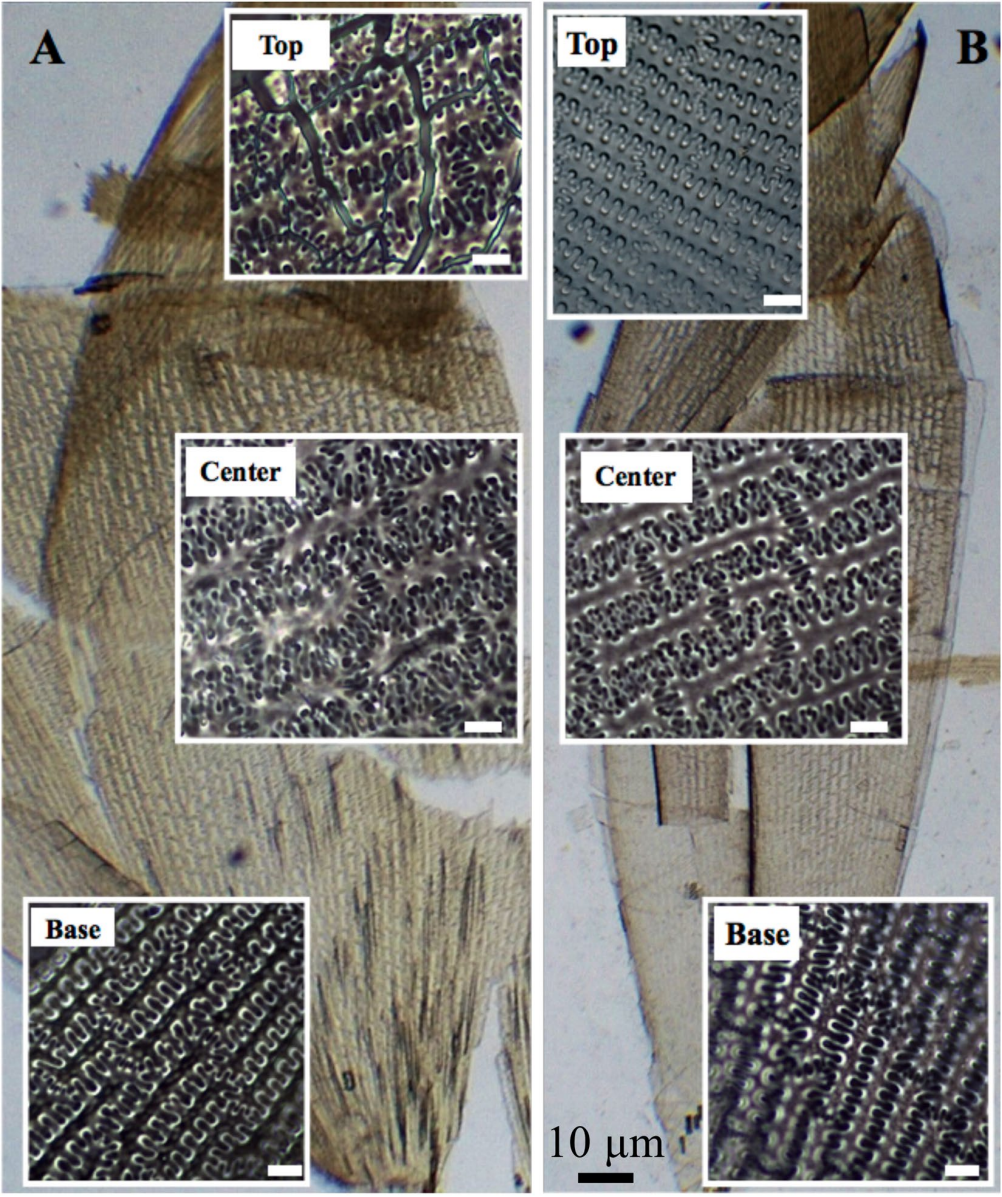
Comparison of silicified patterns of epidermal long cells between domesticated and wild broomcorn millet. (**A**) *Panicum ruderale*, (**B**) *Panicum miliaceum*.

Figure 4 shows the identical features of undulating patterns of epidermal long cells present in the upper lemma and palea in both domesticated broomcorn millet and *P*. *ruderale*; they all exhibit undulating patterns of ηI, ηII and ηIII across the husk as defined by Lu *et al*.^13^. The undulations tend to increase in highly sinuous variation towards the central part of the lemma and palea. The different η-undulated regular patterns in general vary gradually across different parts from the base and top (η I), to the side (ηII), and to the center (η III) of the lemma and palea (Fig. 4).

**Figure 5:**
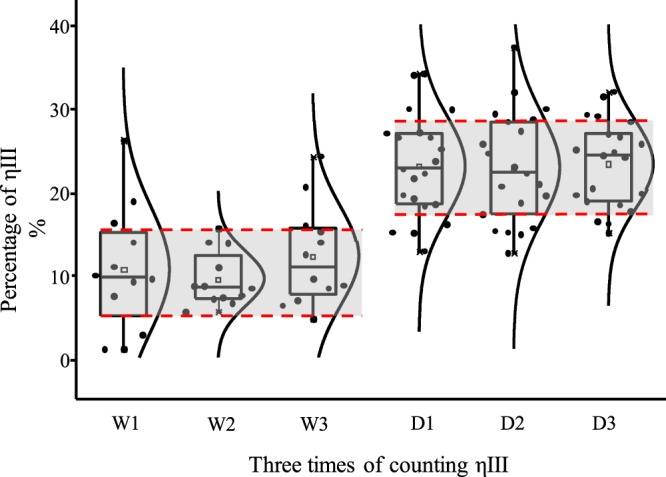
The distribution for the percentage of ηIII in 12 *Panicum ruderale* and 21 *Panicum miliaceum* in the three counts. D1/W1 = 20 pieces of phytoliths were counted regardless of size; D2/W2 = 20 pieces of phytoliths between 125 μm and 375 μm were counted; D3/W3 = 20 pieces of phytoliths both between 125 μm and 375 μm and containing 30 single undulating patterns were counted. The distribution curves are shown on the right of the boxes. The empty squares at the center of the box refer to the mean value of each count. The red dashed lines refer to the outermost quartiles among the three counts, i.e., lower quartiles of D2 and W1, upper quartiles of D2 and W3. D refers to *Panicum miliaceum*; W refers to *Panicum ruderale*.

We statistically analyzed the percentage of ηIII in all counted undulated patterns (ηI, ηII, and ηIII). The percentage of ηIII in both domesticated millet and *P*. *ruderale* in all three counts exhibit normal distributions (Fig. 5). The mean values of the three counts ranged from 9.5% to 12.2% in *P*. *ruderale* and 22.8% to 23.3% in domesticated broomcorn millet, indicating that the distributions of ηIII percentage data for *P*. *ruderale* (Fig. 5, W1–3) and domesticated broomcorn millet (Fig. 5, D1–3) were relatively concentrated within each group regardless of counting method and phytolith size. The mean values of all three counts were 10.8% ± 5.8% (SD; n = 36) and 23.0% ± 5.9% (SD) (n = 63), respectively. Notably, the highest and lowest quartiles among the three counts of *P*. *ruderale* and domesticated broomcorn millet were 5.1–15.5% and 17.2–28.3% without any overlap. The results show that ηIII percentages in domesticated broomcorn millet are markedly higher than those from *P*. *ruderale*, suggesting that the undulated patterns of epidermal long cells in the upper lemma and palea could be used to distinguish between the two species.

**Figure 6:**
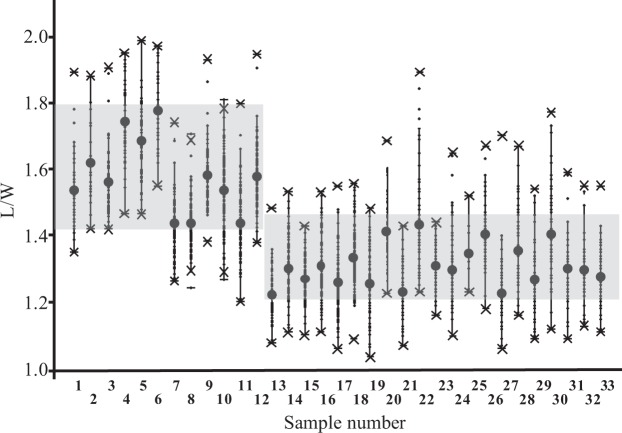
Comparisons of L/W ratio of grains. (1–12) *Panicum ruderale*, (13–33) *Panicum miliaceum*. The gray zones indicate the intervals of the biggest and smallest mean value of L/W in the two species.

The L/W ratio describes the shape, especially the roundness, of grains. The mean L/W of *P*. *ruderale* ranges from 1.44 to 1.78, whereas the ratio of domesticated millet ranges from 1.23 to 1.44 (Fig. 6). This shows that grains of domesticated broomcorn millet have rounder (i.e., fatter and wider) shape on average than *P*. *ruderale*.

**Figure 7:**
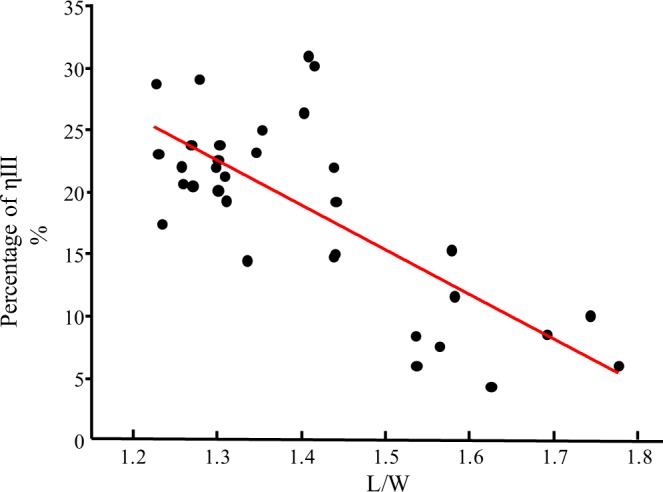
Correlation between the L/W ratio of grains and the ηIII percentage. Linear fit line is shown in red.

A Pearson test was performed on the mean values of each three counts of the ηIII percentage and ratio L/W. The result shows a significant coefficient of −0.749 with the Adj. R^2^ of 0.547 between L/W and ηIII percentage (Table 2), indicating that the ηIII percentage increases with a corresponding decrease in the L/W ratio (Fig. 7).

**Table 2:** Pearson correlation test between the percentages of ηIII and L/W ratio of grains.

		Percentage of ηIII	L/W
Percentage of ηIII	Pearson Correlation	1	−0.749^**^
	Sig. (2-tailed)		0.000
	N	33	33
L/W	Pearson Correlation	−0.749^**^	1
	Sig. (2-tailed)	0.000	
	N	33	33

**Correlation is significant at the 0.01 level (2-tailed).

**Figure 8:**
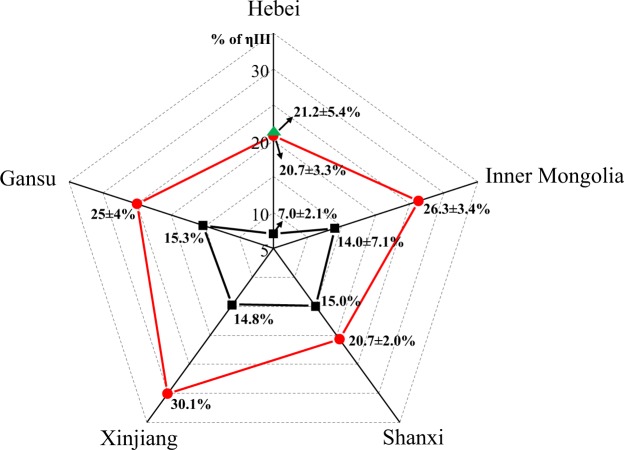
Comparison of ηIII percentages in samples from different climates from semi-humid to arid regions of north China. Red round labels represent *Panicum miliaceum*; black squares represent *Panicum ruderale*; green triangles represent *Panicum miliaceum* originating in Yangyuan County, Hebei Province. Individual data points are the mean values of all three counts.

The percentage of ηIII in samples grown in regions of similar climate have further been compared. The percentage of ηIII in *P*. *ruderale* range from 7.0% to 14.0%, while in *P*. *miliaceum*, percentages range from 20.7% to 30.1%. This shows that regardless of climate region, the percentages of ηIII in *P*. *ruderale* remain markedly lower than those from domesticated landraces. Particularly, six *P*. *ruderale* and two *P*. *miliaceum* samples grown in the same location in Yangyuan County, Hebei Province, exhibited large differences in the mean percentage of ηIII – 7.0% ± 2.1% and 21.2% ± 5.4%, respectively (Fig. 8) – despite the fact that they were grown under the same environmental conditions.

## Discussion

Plant domestication is an evolutionary process that involves a series of profound morphologic and genetic changes^22–25^. Changes in grain size, usually manifesting as a reduction in L/W ratio and greater grain width, are a well-recognized aspect of domestication syndrome in cereals and other seed crops^23,26,27^. Our results show domesticated millet grains have a lower L/W ratio (fatter grains) than that of *P*. *ruderale*, while the percentage of ηIII from domesticated millet is higher than from *P*. *ruderale*. The high correlation between the L/W ratio and the mean value of ηIII percentage suggests that the change in L/W is one of the main factors accounting for the changes in ηIII percentage in epidermal long cells. We speculate that this is because the domestication process enlarges the millet grain width, thus increasing the area of the central part of the husk and inducing the epidermal long cells within the lemma and palea to expand accordingly, resulting in a higher proportion of ηIII.

Phytoliths in the lemma and palea are replicas of epidermal long cells, and their size and undulated patterns change according to the size and shape of seeds. Compared to the top and base of the husk, the central part of the husk is more expanded and rounded. Accordingly, the undulated patterns have the most sinuous type and the largest size, such as ηIII and ΩIII types, as shown in common millet, foxtail millet and green foxtail^13,15^. As Figs 3 and 4 illustrate, seeds of *P*. *ruderale* are not only smaller and thinner than those of domesticated millets, indicating the central part of the husk of *P*. *ruderale* is less expanded, but also significantly correlate with the proportion of ηIII type in the lemma and palea, thus causing the percentage of ηIII in *P*. *ruderale* to be lower than that in the domesticated type.

*P*. *ruderale* has been considered either the wild ancestor or a wild/feral type derived from back-mutation from the domesticated broomcorn millet^28–30^. Generally, feral derivatives of crop varieties may show a similar phenotype to that of the crop ancestor, e.g., thinner and smaller seeds, and shattering^30,31^. Thus, our quantitative data suggest that the direct ancestor of *P*. *miliaceum* may have a lower proportion of ηIII type in the lemma and palea. Owing to the significant correlation between phytolith pattern and seed shape mentioned above, in the absence of exact wild ancestors of broomcorn millet, the characterization of phytolith differences between *P*. *ruderale* and *P*. *miliaceum* thus becomes an alternative approach to provide insight into origin of broomcorn millet that has long been ignored and unexplored.

Analysis of various heritability studies indicates that grain size is affected by both genetic and environmental factors, but that the contribution of heritable genetic traits is greater than the variation attributable to environmental differences^32^. In this paper, we compared the percentage of ηIII from the samples in different originated provinces from semi-humid to arid regions of north China. As Fig. 8 shows, irrespective of climatic region, the percentages of ηIII of *P*. *ruderale* are always lower than those from domesticated millet. In particular, the percentages of ηIII from *P*. *ruderale* and *P*. *miliaceum* samples grown in the same location of Yangyuan County are significantly different and can be distinguished within error range. These quantitative data confirm that environmental conditions may have less effect than genetic variation. Combined with Fig. 4, our data suggest that the percentages of ηIII are not associated with environmental conditions, but significantly correlate with the L/W ratio of grains. This indicates that the percentage of ηIII is a useful parameter facilitating the differentiation of *P*. ruderale and *P*. *miliaceum*.

**Figure 9:**
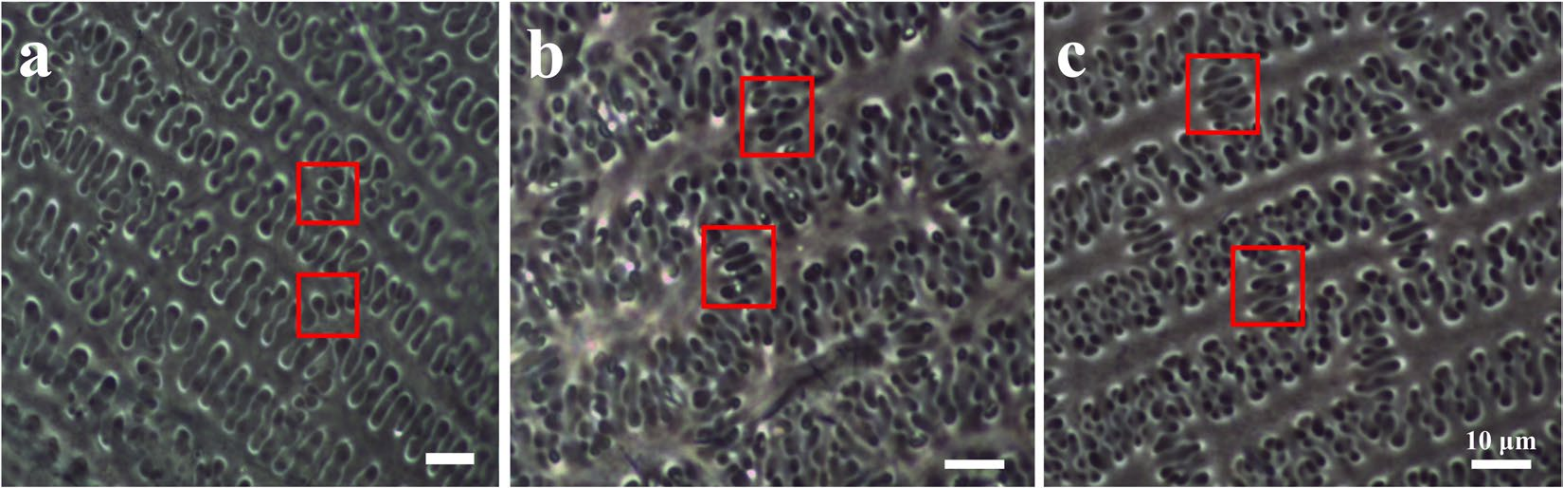
Comparison of silicified patterns of epidermal long cells. (**a**) *Panicum repens*, (**b**) *Panicum ruderale*, and (**c**) *Panicum miliaceum*. The junctions of epidermal long cell are shown in red squares.

Former phylogenetic study indicated that the allotetraploid origin of *P*. *miliaceum*, with the maternal ancestor being *P*. *capillare* (or a close relative) and the other genome being shared with *P*. *repens*^33^. Thus, in order to develop a framework to prevent the misidentification of *P*. *ruderale*, *P*. *miliaceum*, and *P*. *repens*, we further examined the silicon structure patterns in the lemma and palea from the inflorescence bracts in modern *P*. *repens*. The results show that the η type of epidermal long cells also occur in *P*. *repens*, which is identical with *P*. *ruderale* and *P*. *miliaceum*, whereas phytoliths exhibit the ‘wave-type’ morphology in the junction of the epidermal long cell – this is different from the ‘finger-type’ occurring in the other two species^13^ (Fig. 9). This morphological comparison among the three species indicates that the phenotype of epidermal long cells in lemma and palea in similar in *P*. *ruderale* and *P*. *miliaceum*, and suggests that the combination of η type of epidermal long cells plus ‘finger-type’ morphology the junctions of epidermal long cell may be peculiar to *P*. *ruderale* and *P*. *miliaceum*. However, owing to the absence of *P*. *capillare*, which is a native plant to most of North America^34^, we only compared the epidermal long cells from *P*. *repens*, and additional investigation of *Panicum* species are needed to confirm the observations.

In conclusion, regardless of climate region, the percentage of ηIII patterns in *P*. *miliaceum* is markedly higher than the one from *P*. *ruderale*. Because of the significant negative correlation between ηIII pattern percentage and the ratio of grain length/width, in the absence of exact direct wild ancestors of broomcorn millet, the characterization of phytolith differences between *P*. *ruderale* and *P*. *miliaceum* thus becomes an alternative approach to provide insight into origin of broomcorn millet.

## Data Availability

The datasets generated during the current study are available from the corresponding author on reasonable request.
